# Prediction of the morbidity and mortality rates of COVID-19 in Egypt using non–extensive statistics

**DOI:** 10.1038/s41598-023-36959-8

**Published:** 2023-06-21

**Authors:** Hayam Yassin, Eman R. Abo Elyazeed

**Affiliations:** Physics Department, Faculty of Women for Arts, Science and Education, Ain Shams University, Cairo, 11577 Egypt

**Keywords:** Physics, Statistical physics, thermodynamics and nonlinear dynamics

## Abstract

Non–extenstive statistics play a significant role in studying the dynamic behaviour of COVID-19 to assist epidemiological scientists to take appropriate decisions about pandemic planning. Generic non–extensive and modified–Tsallis statistics are used to analyze and predict the morbidity and mortality rates in future. The cumulative number of confirmed infection and death in Egypt at interval from 4 March 2020 till 12 April 2022 are analyzed using both non–extensive statistics. Also, the cumulative confirmed data of infection by gender, death by gender, and death by age in Egypt at interval from 4 March 2020 till 29 June 2021 are fitted using both statistics. The best fit parameters are estimated. Also, we study the dependence of the estimated fit parameters on the people gender and age. Using modified–Tsallis statistic, the predictions of the morbidity rate in female is more than the one in male while the mortality rate in male is greater than the one in female. But, within generic non-extensive statistic we notice that the gender has no effect on the rate of infections and deaths in Egypt. Then, we propose expressions for the dependence of the fitted parameters on the age. We conclude that the obtained fit parameters depend mostly on the age and on the type of the statistical approach applied and the mortality risk increased with people aged above 45 years. We predict - using modified–Tsallis - that the rate of infection and death in Egypt will begin to decrease till stopping during the first quarter of 2025.

## Introduction

Coronavirus was discovered in the city of Wuhan, China^1^ and registered to have the first case of it on the day of December 12th, 2019^1^. This extremely deadly virus was officially named by the world health organization (WHO) as COVID-19 - coronavirus disease 2019 - and it has been noted to attack the respiratory system to the point it resulted in a myriad of infections and countless deaths. Because of how rapidly the virus has been spreading throughout world and entering Egypt also, the Egyptian government had sought numerous ways to decrease the effect of the virus on its citizens as much as possible. The first case to appear in Africa happened to be in Egypt and the virus was recorded to have infected a Chinese person on February 14th, 2020^2^.

The statistical mechanics is the most fundamental field of physics, which applies the probability theory to the realization of the thermodynamic behavior of the systems consisted of large number of particles^3^. The statistical mechanics has helped to solve many problems in science like physical and chemical approaches, bio–mechanics, engineering, computational neuroscience^4–10^. Furthermore, it can be used as a tool for describing the dynamic behaviour of COVID-19^11–15^.

Numerous models have been used to study COVID-19. For example, growth models, differential equations (DE)^16–26^. Also, applications, limitations, and potentials of mathematical models for COVID-19 have been considered by Wang^27^. Various mathematical models were suggested to investigate the outbreak of COVID-19 in Wuhan, China^28,29^. Ivorra et al.^30^ have analyzed the spread of the COVID-19 by developing a mathematical model. Zeb et al.^31^ have utilized a mathematical model for studying the isolation class of COVID-19. Rujira Ouncharone et al. studied a nonlinear mathematical model that addresses the transmission dynamics of COVID-19^32^. Amar Nath Chatterjee et al. proposed a mathematical model to examine the outcome of adaptive immune responses to viral mutation to control disease transmission^33,34^. Many authors have done work on fractional differential equations^35–38^. Also, Anum Shafiq et al. estimated the COVID-19 mortality rates by using artificial neural network (ANN) modeling and maximum likelihood estimation and examined the applicability of ANN models in a study of COVID-19 mortality rates^39^. The COVID-19 mortality rate was also studied using a new alpha power conversion and Gumbel Type-II distribution to propose a unique statistical model^40^. Ahmad Ghanbari et al.^4,11^ proposed a non–extensive entropy–based model to give a log–normal distribution for the spread rate of COVID-19 data over different range of times.

In this paper, we use statistical methods to study COVID-19 in Egypt as we have large amount of data from Central Agency for Public Mobilization and Statistics^41^ and WHO Egypt^42^ to be more effective in understanding Egyptian's circumstances with reference to gender and age. Here, we utilize modified–Tsallis and generic non–extensive statistics. The study aims to analyze the morbidity and mortality rates of COVID-19 in Egypt and predict these rates in future.

## Theoretical approaches

The pandemic data caused by the novel COVID-19 in Egypt have been analyzed by two non–extensive statistical models - modified–Tsallis and generic non–extensive statistics - to predict the active case of the infection and death people in future. We use the data published daily by the Egyptian Ministry of Health and Population - Central Agency for Public Mobilization and Statistics - and World Health Organization Egypt^41,42^. Both non–extensive statistical models are fitted with the confirmed data for obtaining the value of the parameters by using MATLAB software.

### Generic statistics

In generic non–extensive statistics, the partition function reads^43,44^

1 $$\begin{aligned} \ln \, Z(\eta ,\alpha )= & {} \pm \sum _i\, C_i \int _0^{\infty }\, \ln \left[ 1\pm \varepsilon _{c,d,r}(x_i)\right] \; d^3\, t, \end{aligned}$$

where $C_i=\frac{V\, g_i}{(2\, \pi )^3}$ is the normalization constant, $x_i=[\alpha _{i}-E_i]/\eta$ with $E_i(t)=\sqrt{t^2+\zeta ^2}$; the dispersion relation of *i*–th object, *t* is the time in days, $\alpha , \eta , \zeta$ are free parameters. The extended exponential function $\varepsilon _{c,d,r}(x_i)$ is given as^45,46^

2 $$\begin{aligned} \varepsilon _{c,d,r}(x)=\exp \left[ \frac{-d}{1-c} \left( W_k\left[ B\left( 1-\frac{x}{r}\right) ^{\frac{1}{d}}\right] -W_k[B]\right) \right] , \end{aligned}$$

where $W_k$ is the Lambert W-function which has real solutions at $k=0$ with $d\ge 0$ and at $k=1$ with $d<0$,

3 $$\begin{aligned} B=\frac{(1-c)r}{1-(1-c)r} \exp \left[ \frac{(1-c)r}{1-(1-c)r}\right] , \end{aligned}$$

with $r=[1-c+c\,d]^{-1}$ and (*c*, *d*) refer to the non–extensive statistical nature of the underlying system. Thus, the total number of objects in the system^7,47^ can be determined as

4 $$\begin{aligned} N_i= & {} C_i \int _0^\infty \frac{ \varepsilon _{c,d,r}(x_i) \; W_0\left[ B(1-\frac{x_i}{r})^{\frac{1}{d}}\right] }{(1-c)\left[ 1\pm \varepsilon _{c,d,r}(x_i)\right] \left( r-x_i\right) \left( 1+W_0\left[ B(1-\frac{x_i}{r})^{\frac{1}{d}}\right] \right) } d^3 t. \end{aligned}$$

The corresponding distribution is given as

5 $$\begin{aligned}{} & {} P(t,c,d,\alpha ,\eta ,\theta ,\zeta )=\left( t\; \theta \; \eta \; \sqrt{t^2+\zeta ^2} \right) \frac{\varepsilon _{c,d,r}\left( \frac{\alpha -\sqrt{t^2+\zeta ^2}}{\eta }\right) }{(1-c)\left[ 1\pm \varepsilon _{c,d,r}\left( \frac{\alpha -\sqrt{t^2+\zeta ^2}}{\eta }\right) \right] } \times \nonumber \\{} & {} \frac{W_0\left[ B\left( 1-\frac{(\alpha -\sqrt{t^2+\zeta ^2})}{r \eta }\right) ^{\frac{1}{d}}\right] }{\left( r \eta -\alpha +\sqrt{t^2+\zeta ^2}\right) \left( 1+W_0\left[ B\left( 1-\frac{(\alpha -\sqrt{t^2+\zeta ^2})}{r \eta }\right) ^{\frac{1}{d}}\right] \right) }.  \end{aligned}$$

### Modified–Tsallis statistics

The distributions of the cumulative numbers of confirmed infection and death can be described using the modified–Tsallis statistic. The modified–Tsallis distribution^48,49^ is given by

6 $$\begin{aligned} p(t,n, \beta , \theta , \beta ,\zeta )= t \theta \left[ \exp \left( \frac{-\gamma \, \beta \, t}{n \eta }\right) + \frac{\gamma \,\left( \sqrt{t^{2}+\zeta ^{2}}\right) }{n \eta }\right] ^{-n}, \end{aligned}$$

Here, $\beta$ is the average transverse velocity of the system^49^ and $\gamma$ is given by

7 $$\begin{aligned} \gamma = \frac{1}{\sqrt{1-\beta ^2}}, \end{aligned}$$

The power of the non-extensivity of the system *n* is related to the Tsallis parameter *q* as $n = \frac{1}{q-1}$. Smaller values of *n* correspond to larger values of *q* which indicating the non-equilibrium state for the system. Also, *n* can be known as the entropic parameter. Both *q* and *n* have been alternately utilized in the Tsallis distributions^7,50,51^.

In the section that follows, we introduce results on the distributions of the cumulative numbers of confirmed infection and death cases in Egypt at interval from 4 March 2020 till 12 April 2022^42^. Also, we introduce results on the distributions of the cumulative number of confirmed infections by gender, death by gender, and death by age in Egypt at interval from 4 March 2020 to 29 June 2021^41^. All results are calculated within modified–Tsallis and generic non–extensive statistics.

**Figure 1:**
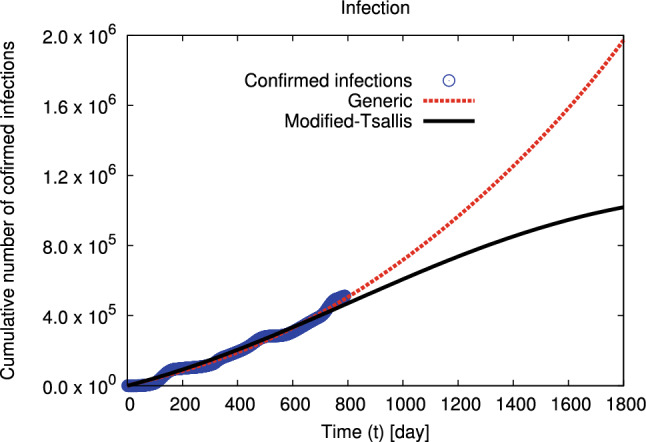
The distributions of the cumulative number of confirmed infections in Egypt, at interval from 4 March 2020 till 12 April 2022 (symbols)^42^ are fitted to generic non–extensive statistics and modified–Tsallis (dashed and solid curves) using Eqs. (5, 6), at interval from first confirmed day of infection to 1800 days (4 February 2025).

**Figure 2:**
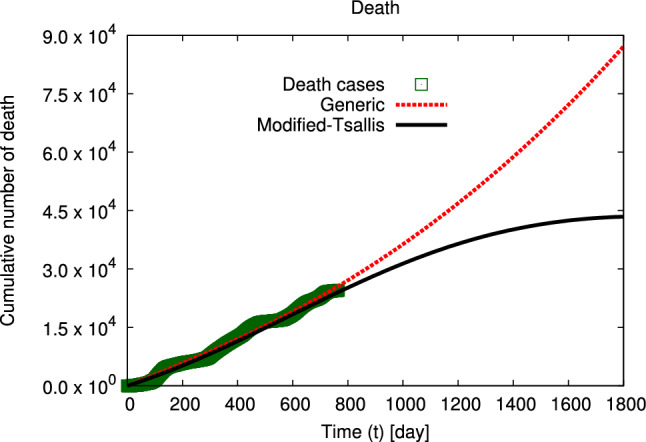
The distributions of the cumulative number of confirmed deaths in Egypt, at interval from 12 March 2020 till 12 April 2022 (symbols)^42^ are fitted to generic non–extensive statistics and modified–Tsallis (dashed and solid curves) using Eqs. (5, 6), at interval from 12 March 2020 to 1800 days (12 February 2025).

## Results

**Figure 3:**
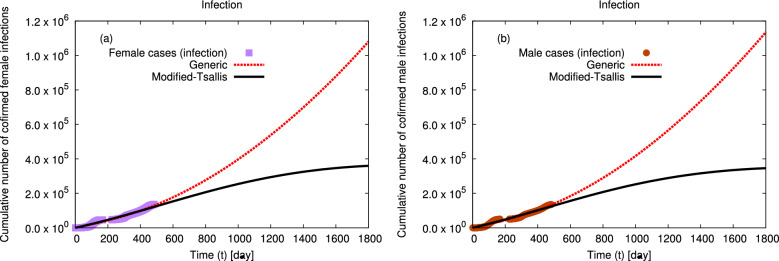
The distributions of the cumulative number of confirmed infection (**a**) for female and (**b**) for male, in Egypt, at interval from 4 March 2020 till 29 June 2021 (symbols)^41^ are fitted to generic non–extensive statistics and modified–Tsallis (dashed and solid curves) using Eqs. (5, 6), since first confirmed case to 1800 days (4 February 2025).

**Figure 4:**
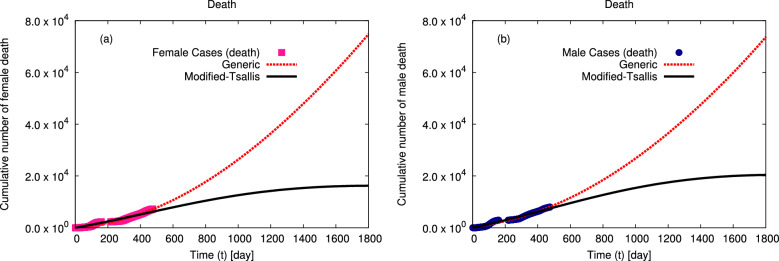
The distributions of the cumulative number of confirmed death (**a**) for female and (**b**) for male, in Egypt, at interval from 4 March 2020 till 29 June 2021 (symbols)^41^ are fitted to generic non–extensive statistics and modified–Tsallis (dashed and solid curves) using Eqs. (5, 6), at interval from the day of first confirmed death case to 1800 days (12 February 2025).

**Figure 5:**
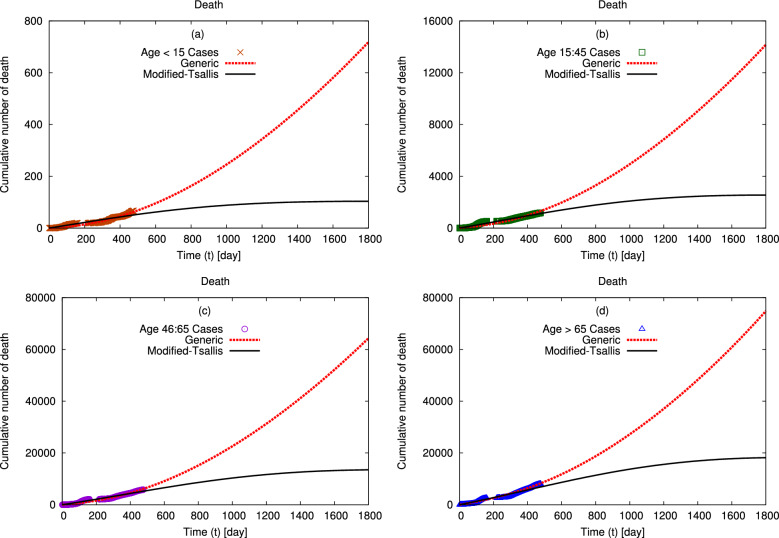
The distributions of the cumulative number of confirmed deaths in Egypt, at interval from 4 March 2020 till 29 June 2021 (symbols)^41^ are fitted to generic non–extensive and modified–Tsallis statistics (dashed and solid curves) using Eqs. (5, 6). Panels (**a**–**d**) depicts the impacts of changing age interval from $< 15$ year to $15-45$ year to $46-65$ year and to $>65$ year, respectively. The corresponding fit parameters are given in Figs. 6, 7, 8 and 9.

The distributions of the cumulative numbers of confirmed infection and death cases in Egypt at interval from 4 March 2020 till 12 April 2022^42^ are fitted using modified–Tsallis and generic non–extensive statistics and shown in Figs. 1 and 2, respectively. The goodness of the statistical fits is listed in Table 1. The distributions of the cumulative numbers of confirmed infection by gender, death by gender, and death by age in Egypt at interval from 4 March 2020 to 29 June 2021^41^ are fitted using modified–Tsallis and generic non–extensive statistics and shown in Figs. 3, 4, and 5, respectively. The goodness of the statistical fits is listed in Tables 1 and 4.

**Table 1:** The qualities ($\chi ^2$) of the modified–Tsallis and generic non–extensive statistical fits for the distributions are determined for all infection and death, infection by gender, and death by gender at interval start from 4 March 2020 till 29 June 2021^41^.

Type	$\chi ^2$ (Modified–Tsallis)	$\chi ^2$ (Generic)
Infection (all)	0.1228	0.5881
Death (all)	0.0948	0.3227
Infection (female)	0.3642	0.9065
Infection (male)	0.3715	0.8389
Death (female)	0.3463	1.421
Death (male)	0.2529	0.8549

Figures 1 and 2 depict the distribution of the cumulative number of confirmed infection and death in Egypt, respectively. The symbols indicate to the confirmed data in Egypt from the beginning of spreading the disease till 12 April 2022^42^. Solid and dashed curves refer to our calculations using modified–Tsallis statistics (Eq. (6)) and generic non–extensive statistics (Eq. (5)), respectively. The fitting parameters by both statistics are presented in Tables 2 and 3. Our calculations agree well with the confirmed data in Egypt at the studied interval (4 March 2020 till 12 April 2022). Additionally, our calculations using modified–Tsallis statistic predict that the rate of spreading of infection and the death rate will decrease till to be approximately stopped after 1800 days from the first confirmed case of infection. While our results using generic non–extensive statistics predict that both infection and death rates increase continuously but by a slowly rate.

Figures 3 and 4 present the distributions of the cumulative number of confirmed (a) female and (b) male infection and death in Egypt, respectively. The symbols refer to the confirmed data for both genders in Egypt from the confirmed infection and death cases till 29 June 2021^41^. Our calculations using modified–Tsallis statistics and generic non–extensive statistics are represented by solid and dashed curves, respectively. The obtained fit parameters by both statistics are given in Tables 2 and 3. Our calculations agree well with the confirmed data. Additionally, our calculations using modified–Tsallis statistic predict that the rate of spreading of infection and the death rate will decrease till to be approximately stopped after 1800 days from the first confirmed case of infection. While our results using generic non–extensive statistics predict that both infection and death rates increase continuously but by a slowly rate.

**Table 2:** The various fit parameters obtained from modified–Tsallis statistics, Figs. 1, 2, 3 and 4.

Type	*n*	$\beta$	$\theta$	$\eta$	$\zeta$
All infection	$4.0818\pm 0.0144$	$0.7266\pm 0.001$	$11106.5\pm 32.2$	$668.827\pm 0.8925$	$2344.16\pm 2.878$
Female infection	$3.8286\pm 0.0563$	$0.545\pm 0.0073$	$1683.843\pm 9.964$	$663.843\pm 2.574$	$1546.31\pm 5.631$
Male infection	$2.6319\pm 0.0274$	$0.5344\pm 0.0076$	$1600.16\pm 8.406$	$601.918\pm 2.358$	$1500.34\pm 5.574$
All death	$3.5046\pm 0.0085$	$0.6999\pm 0.0009$	$547.004\pm 1.121$	$498.346\pm 0.4999$	$1855.93\pm 1.759$
Male death	$3.203\pm 0.0159$	$0.4748\pm 0.0061$	$314.064\pm 1.628$	$383.334\pm 1.008$	$1818\pm 4.597$
Female death	$2.9723\pm 0.0175$	$0.4705\pm 0.0084$	$299.997\pm 2.114$	$338.632\pm 1.213$	$1800.3\pm 6.235$

**Table 3:** The various fit parameters obtained from generic non–extensive statistics, Figs. 1, 2, 3 and 4.

Type	*c*	*d*	$\alpha$	$\theta$	$\eta$	$\zeta$
All infection	$1.24\pm 0.004$	$1.987\pm 0.005$	$2407.06\pm 5.9$	$147.841\pm 0.34$	$7.009\pm 0.014$	$560.584\pm 3.3$
Female infection	$1.227\pm 0.001$	$1.974\pm 0.009$	$2946.96\pm 14.8$	$131.615\pm 0.65$	$5.224\pm 0.023$	$489.057\pm 4.43$
Male infection	$1.154\pm 0.001$	$1.951\pm 0.008$	$3246.96\pm 14.8$	$121.612\pm 0.55$	$4.182\pm 0.017$	$477.741\pm 4.01$
All death	$1.18\pm 0.003$	$1.969\pm 0.003$	$2977.08\pm 6.5$	$163.613\pm 0.32$	$0.328\pm 0.001$	$1084.98\pm 3.5$
Male death	$1.16\pm 0.005$	$1.949\pm 0.006$	$3049.2\pm 12.8$	$159.419\pm 0.66$	$0.289\pm 0.001$	$407.965\pm 3.49$
Female death	$1.101\pm 0.001$	$1.941\pm 0.006$	$3649.3\pm 15.0$	$127.962\pm 0.54$	$0.252\pm 0.001$	$249.634\pm 3.55$

Tables 2 and 3 present the various fit parameters obtained from fitting the confirmed data of infections and deaths within gender at the studied interval using modified–Tsallis and generic non–extensive statistics, respectively. For both statistics, we observe that all constants increase with the increasing in the confirmed number of infection or death in order male to female to all infections (female to male to all deaths) except $\alpha$. Furthermore, we note that the fit parameters for female infections are greater than ones for male infection except $\alpha$. While the opposite behaviour is noticed for death fit parameters where the fit parameters from death in male are greater than the ones for death in female except $\alpha$. Furthermore, using modified–Tsallis statistic we find that the rate of infection in female is more than the one in male while the rate of death in male is greater than the one in female due to the differences between females and males in the immune response^52,53^ - X chromosome and sex hormones - to infection with the coronavirus and inflammatory diseases and also, the levels of activation of immune cells in females are higher than in males, which leads to female immunity being able to eliminate the disease or reduce its impact, unlike males, as shown in Figs. 3 and 4. Our results agree with previous study^54^. However, within generic non–extensive statistic we notice that the gender has no effect on the rate of infections and deaths in Egypt.

**Table 4:** The qualities ($\chi ^2$) of the modified–Tsallis and generic non–extensive statistical fits for the distributions are determined for death by age at interval start from 4 March 2020 till 29 June 2021^41^.

Age	Age interval *A*	$\chi ^2$ (Modified–Tsallis)	$\chi ^2$ (Generic)
$<15$	1	0.502	0.5655
15 : 45	2	0.2123	0.662
46 : 65	3	0.2551	0.4508
$>65$	4	0.3176	0.4164

**Figure 6:**
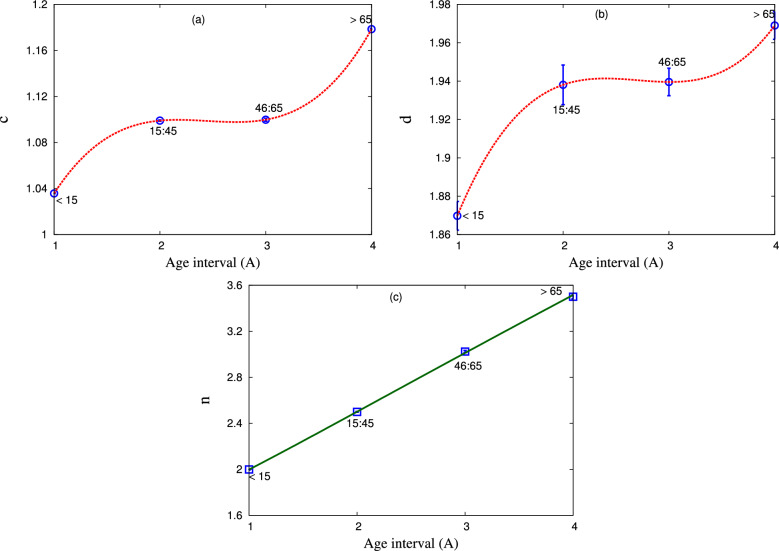
The non–extensive parameters *c*, *d*, and *n* as obtained from the statistical fits from generic non–extensive and modified–Tsallis statistics for the cumulative number of death cases at different age intervals. The solid curves indicate to the proposed expressions for the dependence of non–extensive parameters on the death age interval.

Figure 5 shows the distributions of the cumulative number of confirmed deaths in Egypt at age intervals (a) less than 15 year, (b) $15-45$ year, (c) $46-65$ year, and (d) greater than 65 years. The symbols represent the confirmed death for each age interval in Egypt from the beginning of death till 29 June 2021^41^. Dashed and solid curves are the generic non–extensive and modified–Tsallis distributions fitted to the confirmed data, respectively. Our calculations using Eqs. (5,6) agree well with the confirmed data. We notice that the rate of death increases with the age. Moreover, our calculations using modified–Tsallis statistic predict that the rate of spreading of infection and the death rate will decrease till to be approximately stopped after 1800 days from the first confirmed case of infection. While our results using generic non–extensive statistics predict that both infection and death rates increase continuously but by a slowly rate.

As seen from Tables 1, 4, the value of $\chi ^2$ is small which confirming the goodness of the fitting quality. Especially, the modified–Tsallis statistics shows excellent agreement with the confirmed data of both infection and death in Egypt in the studied interval.

**Figure 7:**
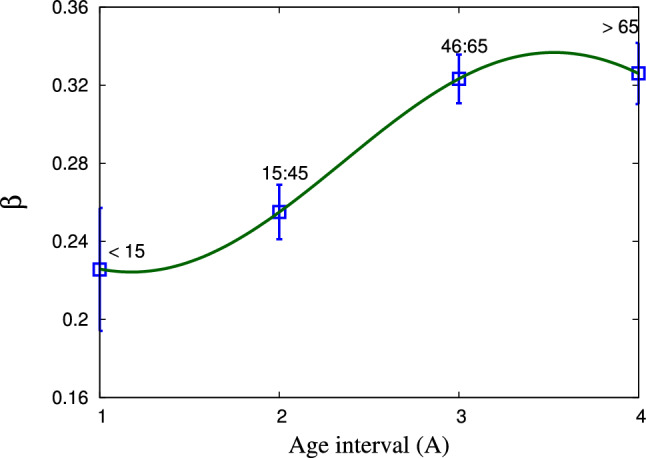
The dependence of the fit parameter $\beta$ as deduced from the statistical fit within modified–Tsallis statistic on the age of death cases in Egypt. The curve represents the proposed expression for the studied dependence.

**Figure 8:**
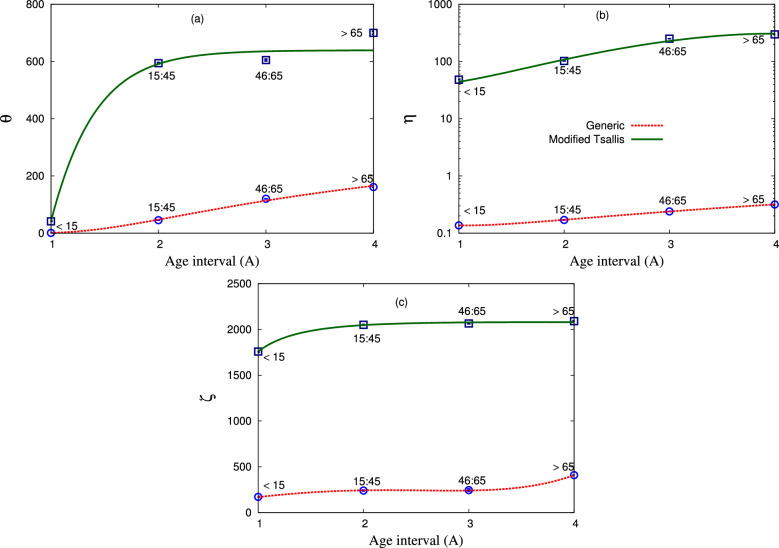
The fit parameters $\theta$, $\eta$, and $\zeta$ as obtained from the statistical fit within both statistics on the age of death cases in Egypt. Square and circle symbols refer to the obtained fitting parameters within modified–Tsallis and generic non–extensive statistics, respectively. While the solid and dotted curves represent the corresponding proposed expressions for this dependence, respectively.

**Figure 9:**
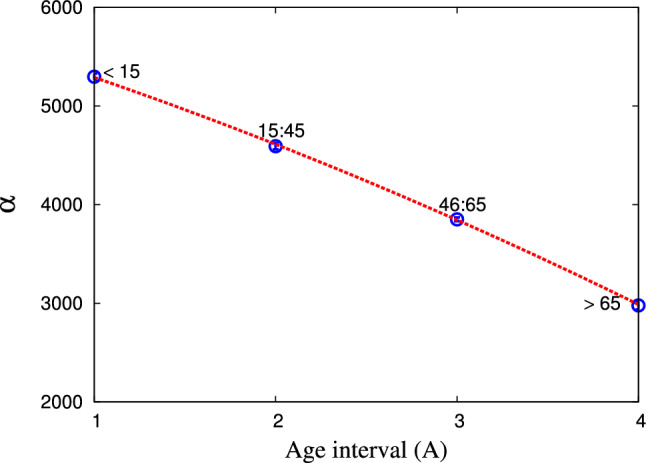
The dependence of the fit parameter $\alpha$ as deduced from the statistical fit within generic non–extensive statistic on the age of death cases in Egypt. The curve represents the proposed expression for the studied dependence.

In the following section, we will propose expressions relating the dependence of the resulting fit parameters on the age interval *A*. Fit parameters are obtained from the comparison between our calculations using modified–Tsallis and generic non–extensive statistics and the confirmed death cases in Egypt in the interval from the beginning of death to 29 June 2021^41^. We will debrief various fit parameters from both types of statistics as shown in Figs. 6, 7, 8 and 9.

### Correlations between fit parameters

Figure 6 presents the dependence of the non–extensive parameters *c*, *d*, and *n* on the age interval for the death cases in Egypt which obtained from generic non–extensive and modified–Tsallis statistics in panels (a–c), respectively. Panels (a,b) depict the non–extensive parameters *c*, *d* as a function of the interval age for the death people in Egypt, which have been fitted to generic non–extensive statistic. We observe that both *c* and *d* increase with the increase in the age interval, especially in the young age. Then, approximately saturation values in the interval from $15-65$. After that, *c*, *d* increase again with the increase in age. Panel (c) depicts the dependence of the entropic parameter *n* on the age. We note that *n* increases linearly with the increase in age. So, we conclude that the non-equilibrium state of our system increases with the increase in people age.

Figure 7 depicts the dependence of the fit parameter $\beta$ on the age of death cases in Egypt. We observe that $\beta$ increases with the increase in the age interval till 65 years, while it decreases at age older than 65. Figure 8 shows the fit parameters $\theta$, $\eta$, and $\zeta$ in dependence of the age interval for the death cases in Egypt. The square and circle symbols refer to the obtained fitting parameters within modified–Tsallis and generic non–extensive statistics, respectively. While the solid and dotted curves represent the corresponding - modified–Tsallis and generic non–extensive statistics - proposed expressions for this dependence. Panel (a) depicts the dependence of $\theta$ on *A*. We note a different behaviour of $\theta$ from both statistics. On one hand, $\theta$ using modified–Tsallis statistic increases rapidly with age and after that, $\theta$ increases very slowly with age. On the other hand, $\theta$ within generic non–extensive statistic increases linearly with age.

Figure 8b shows the dependence of $\eta$ on *A* within both statistics. We find that $\eta$ increases with the age using both statistics. The dependence of $\zeta$ on *A* is represented in Fig. 8c. It is noted that $\zeta$ within modified–Tsallis statistic increases with age then becomes nearly constant at old age, while $\zeta$ within generic non–extensive statistic increases gradually with age till interval 46 : 65 then increases rapidly with age greater than 65. We conclude that all previous fit parameters (especially $\theta$, $\eta$, $\zeta$) increase with age due to weak immunity and many diseases associated with age, in addition to the frequent exposure to the virus from more than one source. Figure 9 presents the fitting parameter $\alpha$ which deduced within generic non–extensive statistic as a function of the age of the death’s cases in Egypt. We observe that $\alpha$ decreases with the increase in age.

**Table 5:** The various fit parameters obtained from the proposed expressions within modified–Tsallis statistics, Eqs. (8–12.

Fit parameters	*b*	*f*	*h*
*n*	$2.1878\pm 0.1841$	$1.295\pm 0.0827$	$0.5972\pm 0.028$
$\beta$	$-0.0562\pm 9.73\times 10^{-5}$	$1.3353\pm 0.0006$	$0.2805\pm 7.382\times 10^{-5}$
$\theta$	$4302.96\pm 3.7$	$1.2977\pm 0.0029$	$-3663.96\pm 3.205$
$\eta$	$-0.4191\pm 0.0239$	$10.9566\pm 0.3129$	$33.4786\pm 4.163$
$\zeta$	$7.6219\pm 0.0225$	$0.1376\pm 0.0047$	$0.7217\pm 0.0081$

**Table 6:** The various fit parameters obtained from the proposed expressions within generic non–extensive statistics, Eqs. (10–14).

Fit parameters	*b*	*f*	*h*	*j*
*c*	$0.0234\pm 0.0001$	$-0.1716\pm 0.0001$	$0.4143\pm 0.0001$	$0.77\pm 0.001$
*d*	$0.0158\pm 0.0001$	$-0.1282\pm 0.0001$	$0.3423\pm 0.0001$	$1.64\pm 0.001$
$\alpha$	$-44.5694\pm 0.682$	$-544.401\pm 2.162$	$5878.51\pm 5.9$	−
$\theta$	$1.9999\pm 0.0104$	$-1.2078\pm 0.018$	$8.5904\pm 0.2643$	−
$\eta$	$0.0585\pm 0.0002$	$1.175\pm 0.0023$	$0.0782\pm 0.0006$	−
$\zeta$	$0.9465\pm 0.0013$	$-20.5302\pm 0.0203$	$188.325\pm 0.305$	−

#### Analytical expressions for the resulting fit parameters

Tables 5 and 6 and Figs. 6, 7, 8 and 9 list and present numerous fit parameters which deduced from the fitting of the studied confirmed data of infection and death in Egypt. In the following, we abstract the various dependence of these parameters on the people age.

- Using modified–Tsallis statistics, the dependence of *n* on age interval *A* is given as
  8 $$\begin{aligned} n = (b + A^f)^h, \end{aligned}$$
  where the values of *b*, *f*, and *h* are taken from Fig. 6(c), see Table 5.
- The dependence of $\beta$ on age interval *A* is expressed as
  9 $$\begin{aligned} \beta = b \sin (f A)+ h, \end{aligned}$$
  where the values of *b*, *f*, and *h* are taken from Fig. 7, see Table 5.
- The dependence of $\theta$ on age interval *A* is suggested as
  10 $$\begin{aligned} \theta =\left\{ \begin{array}{cc} b \tan (f A) + h, &{} \texttt {modified-Tsallis} \\ (b- A^f)^h, &{} \texttt {generic} \end{array} \right\} \end{aligned}$$
  where the values of *b*, *f*, and *h* are taken in Fig. 8(a), see Tables 5, 6.
- The dependence of $\eta$ on age interval *A* can be expressed as
  11 $$\begin{aligned} \eta =\left\{ \begin{array}{cc} b A^5+f A^3 + h, &{} \texttt {modified-Tsallis} \\ b A^f + \frac{h}{A}, &{} \texttt {generic} \end{array} \right\} \end{aligned}$$
  where the values of *b*, *f*, and *h* are taken from Fig. 8(b), see Tables 5, 6.
- The dependence of $\zeta$ on age interval is given as
  12 $$\begin{aligned} \zeta =\left\{ \begin{array}{cc} \exp \left[ \frac{b}{A}+f\right] \cos \left[ \frac{h}{A}\right] , &{} \texttt {modified-Tsallis} \\ b A^5+f A^3 + h A, &{} \texttt {generic} \end{array} \right\} \end{aligned}$$
  where the values of *b*, *f*, and *h* are taken from Fig. 8(c), see Tabs. 5, 6.
- Using generic non–extensive statistics, the dependence of *c* and *d* on age interval (*A*) can be expressed as
  13 $$\begin{aligned} c \; \texttt {and} \; d = b A^3+f A^2 + h A + j, \end{aligned}$$
  where the values of *b*, *f*, *h*, and *j* are taken in Fig. 6a,b, see Table 6.
- The dependence of $\alpha$ on age interval *A* is expressed as
  14 $$\begin{aligned} \alpha = b A^2 + f A + h, \end{aligned}$$
  where the values of *b*, *f*, and *h* are taken from Fig. 9, see Table 6.

Therefore, we conclude that the obtained fit parameters depend on the age and on the type of the statistical approach applied.

## Conclusions

We analyzed the pandemic data caused by the coronavirus disease 2019 using modified–Tsallis and generic non–extensive statistics to predict the morbidity and mortality rates in future. Both statistics are fitted with the confirmed data of infection and death cases in Egypt at interval from 4 March 2020 till 12 April 2022^42^. Also, we fit the cumulative number of confirmed infections by gender, death by gender, and death by age in Egypt at interval from 4 March 2020 to 29 June 2021^41^ using both kinds of non–extensive statistics. We have deduced the best fit parameters using MATLAB software. The fit parameters are presented as a function of the people gender and age in infection and(/or) death. Also, we propose expressions for the studied dependence of fitted parameters. Then, we conclude that the obtained fit parameters depend mostly on the age and on the type of the statistical approach applied.

We find that our calculations using modified–Tsallis statistic predict the rate of spreading of infection and the death rate in Egypt will decrease till to be approximately stopped after 1800 days - first quarter of 2025 - from day of the confirmed infection case. While our results using generic non–extensive statistics predict that both infection and death will increase directly with time. Also, within modified–Tsallis statistic, we conclude that the rate of death in male is greater than the death in female due to the differences between females and males in the immune response to infection with the coronavirus and inflammatory diseases and also, the levels of activation of immune cells in females are higher than in males, which leads to female immunity being able to eliminate the disease or reduce its impact, unlike males. Our results agree with previous study^54^.

Furthermore, we have analyzed the dependence of the death rate on the age. We find that the age distribution has a clear impact on the number of deaths. There is a direct correlation between death and advancing age. The older the age, the higher the likelihood of people suffering from chronic diseases such as high blood pressure, heart diseases, diabetes, or cancers, which is reflected in a decrease in immunity. So, the mortality risk increased with those people. People aged above 45 years showed $91.15\%$ of COVID-19-related deaths in Egypt in the studied interval (from 12 March 2020 till 29 June 2021).

In the further work, we will apply the non–extensive statistics on Egypt’s governorates and other countries in the world to investigate the effect of the vaccine on the morbidity and mortality rates of COVID-19.

## Data Availability

The datasets generated during and/or analyzed during the current study are available from the corresponding author on reasonable request.
